# Advanced humidity sensing properties of CuO ceramics

**DOI:** 10.1038/s41598-024-60421-y

**Published:** 2024-04-27

**Authors:** Kaniknun Sreejivungsa, Noppakorn Thanamoon, Nutthakritta Phromviyo, Wirat Jarernboon, Masaki Takesada, Prasit Thongbai

**Affiliations:** 1 Giant Dielectric and Computational Design Research Group (GD–CDR), Department of Physics, Faculty of Science, Khon Kaen University, Khon Kaen, 40002 Thailand; 2 Department of Physics, Hokkaido University, Sapporo, 060-0810 Japan

**Keywords:** CuO, Giant, Colossal permittivity, IBLC, Capacitive humidity sensors, Hysteresis error, Materials science, Physics

## Abstract

This research explores the capacitive humidity sensing properties of CuO ceramic, selected for its simplicity as an oxide and ease of fabrication, in addition to its remarkable dielectric properties. The CuO sample was fabricated by sintering at 980 °C for 5 h. A microstructure with a relative density of 88.9% was obtained. X-ray diffraction confirmed the formation of a pure CuO phase. Broadband dielectric spectroscopy revealed that the observed giant dielectric properties at room temperature (RT) were attributed to extrinsic effects, including the internal barrier layer capacitor and sample-electrode contact effects. A key focus of this study was to examine the giant dielectric properties of CuO ceramic as a function of relative humidity (RH) at RT and frequencies of 10^2^ and 10^3^ Hz. It was observed that the capacitance of CuO continuously increased with rising RH levels, ranging from 30 to 95%. Notably, the maximum hysteresis errors were constrained to 2.3 and 3.3% at 10^2^ and 10^3^ Hz, respectively. Additionally, the CuO ceramic demonstrated very fast response and recovery times, approximately 2.8 and 0.95 min, respectively. The repeatability of the humidity response of the capacitance was also established. Overall, this research highlights the high potential of CuO as a giant dielectric material for application in humidity sensors.

## Introduction

Giant dielectric materials, a groundbreaking class of materials, have attracted significant attention due to their exceptional dielectric properties^1,2^. Characterized by their unusually high relative dielectric permittivity (ε′), these materials, such as CaCu_3_Ti_4_O_12_^3–5^, SrTiO_3_^6–8^, La_2-x_Sr_x_NiO_4_^9^, TiO_2_^10^, NiO^11^, and CuO^12–15^, stand out in the field of electronics. The most captivating feature of these materials is their ability to maintain a high ε′ across a wide range of frequencies. This unique combination of features opens up a wide array of applications, especially in the field of energy storage and capacitors Moreover, their potential in miniaturizing components without compromising performance is leading to advancements in various sectors, including telecommunications, automotive electronics, and even in the development of next–generation computing systems^1,2^. The exploration of giant dielectric materials is not just a scientific pursuit but a step towards a more efficient and miniaturized electronic future.

Among giant dielectric oxides, giant dielectric properties of CuO, a simple oxide, have drawn significant attention in the field of materials science due to their exceptional characteristics and potential applications^13,16^. CuO is known for its exceptionally high ε′ of 10^4^, a property that makes it stand out among conventional dielectric materials. This characteristic is primarily due to its unique electronic structure and the presence of various defects and grain boundaries within its crystalline structure. The internal barrier layer capacitor (IBLC) effect was initially suggested as the origin of the giant dielectric response in CuO^13,14^. However, it was later proposed that the sample-electrode (S–E) interface effect plays a more significant role in influencing this giant dielectric response, rather than the IBLC^16^. CuO stands out not only for its ε′ value but also for its exceptional stability across a vast range of temperatures and frequencies, which enhances its suitability for various applications. Additionally, the non-toxic nature and relative abundance of copper make CuO a more environmentally friendly option compared to other materials with similar properties. Furthermore, the sintering temperature of CuO (approximately 800–980 °C) is much lower than that of other giant dielectric oxides, such as CaCu_3_Ti_4_O_12_ at 1050–1100 °C^3–5,17^, BaTiO_3_ at 1400 °C ^18^, SrTiO_3_ at 1400–1550 °C^6^, La_2-*x*_Sr_*x*_NiO_4_ at 1350–1400 °C^19^, and NiO at 1400–1610 °C^11^, and TiO_2_–based oxides at 1350–1550 °C^10,20,21^.

Recently, the exploration of humidity sensing properties in giant dielectric oxides, particularly those like co–doped TiO_2_, SnO_2_, CaCu_3_Ti_4_O_12_, and related ACu_3_Ti_4_O_12_ compounds, has also opened new avenues in the field of sensor technology^22–28^. These materials are renowned for their exceptional dielectric properties, which are significantly influenced by environmental humidity. This sensitivity to moisture makes them ideal candidates for use in humidity sensors, a critical component in various industrial, environmental, and biomedical applications^29,30^. The growing interest in these giant dielectric oxides for capacitive humidity sensing is driven by their potential to offer more sensitive, reliable, and durable sensors compared to their temperature and chemical stability. Their capability to operate over a wide range of temperatures and environmental conditions further adds to their appeal. The integration of these materials into sensor technology could lead to advancements in climate control systems, environmental monitoring, agricultural production, and healthcare diagnostics.

One of the key parameters for employing giant dielectric oxides in humidity sensors is the hysteresis error (γ_H_)^22,23,26,27,29^. This error arises from the difference between the forward and backward changes in the ε′ or capacitance (C_p_) during the absorption and desorption of H_2_O molecules by giant dielectric oxides. It is crucial to minimize the maximum hysteresis error (γ_H,max_). For instance, the γ_Hmax_ values for CaCu_3_Ti_4_O_12_ were found to range between 9.2 and 20.1%^26^, but Mg-doping in CaCu_3_Ti_4_O_12_ has reduced it to 7.0%^26^. More recently, (Na_1/3_Sr_1/3_Tb_1/3_)Cu_3_Ti_4_O_12_ demonstrated a minimized γ_Hmax_ value of 10.3%^28^. Additionally, 5%(Na, Nb) co–doped TiO_2_ and 10%(In, Nb) co–doped TiO_2_ exhibited low γ_Hmax_ values of 4.2% and 7.3%^22,23^, respectively. However, the sintering temperatures for these TiO_2_–based humidity sensor materials are quite high. Furthermore, the response and recovery times are also crucial parameters. For CaCu_3_Ti_4_O_12_, these times are greater than 10 min^26^, which is relatively long and may not be suitable for optimal performance in humidity sensors.

In this research work, we demonstrated that CuO ceramic is notably effective for humidity sensing, attributed to its simplicity as an oxide, ease of fabrication, and remarkable giant dielectric response. The focus was on the capacitive humidity sensing properties of CuO, which was sintered at 980 ℃ to achieve a microstructure with a relative density of 88.9%. At low frequencies, a significant increase in C_p_ was observed with RH ranging from 30 to 95%. This behavior was further characterized by low hysteresis errors (2.3–3.3%) and rapid response and recovery times (2.8 and 0.95 min, respectively). The repeatability of these responses highlights the potential of CuO as a dielectric material in humidity sensors. These insights not only contribute to a deeper understanding of CuO ceramics but also display its significant potential in advancing sensor technologies, particularly with implications for environmental monitoring applications.

### Experimental method

Copper(II) oxide (CuO) with a purity of 99.9%, obtained from Sigma-Aldrich (St. Louis, MO, USA), was used as the starting raw powder. CuO powder was ball-milled in ethanol (99.9% purity, RCI Labscan, Bangkok, Thailand) for 24 h to obtain fine powder, using ZrO_2_ balls as grinding media. Subsequently, the mixture was heated at 80 °C for 24 h to evaporate the ethanol. The resulting mixed powders were then compressed into pellets. The pellets were sintered in air at 980 °C for 5 h at a heating rate of 5 °C/min, followed by natural cooling in the furnace to room temperature.

The density of the samples was determined using the Archimedes method. The phase composition of the sintered samples was characterized via X–ray diffraction (XRD) using a PANalytical EMPYREAN instrument. XRD patterns were recorded over a 2θ range of 20° to 80°. Quantitative analysis was conducted using the Rietveld method, implemented through the X'Pert HighScore Plus software package provided by PANalytical. The microstructure and elemental distribution within the sintered ceramics were investigated using field emission scanning electron microscopy (FE-SEM, FEI, Helios Nanolab G3CX).

Dielectric properties were assessed using an impedance analyzer (KEYSIGHT E4990A). In preparation for these measurements, both surfaces of each sample were coated with silver paint, which was then dried at 100 °C for 30 min. The ambient temperature and humidity conditions during the measurements were regulated using a temperature–humidity chamber (SH–222, ESPEC). Dielectric responses were recorded under an AC oscillation voltage of 0.5 V, spanning a frequency range from 10^2^ to 10^6^ Hz and a relative humidity (RH) range of 30 to 95%. For temperatures from − 258 to – 20 ℃, a closed–cycle helium cryostat (Daikin Cryo–Kelvin V2O2C5L) was used. Simultaneously, data acquisition was performed using an Agilent 4294A Precision Impedance Analyzer.

## Results and discussion

**Figure 1:**
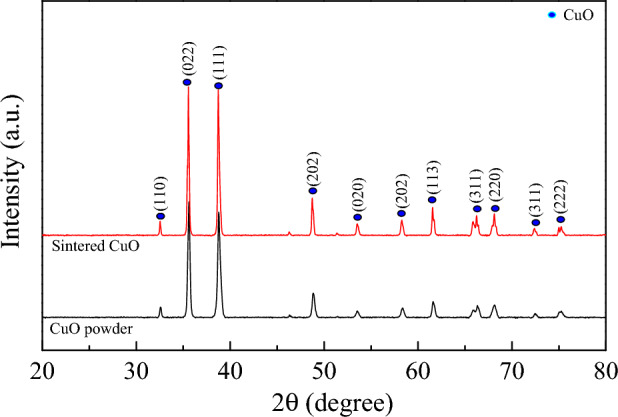
XRD patterns of CuO powder and sintered CuO ceramic.

The XRD patterns of CuO powder and sintered CuO ceramic, presented in Fig. 1, show distinct peaks that are characteristic of the monoclinic phase of CuO, without any signs of impurity phases. This indicates that the synthesized materials are of high purity, which is supported by the absence of unassigned peaks that would typically suggest contamination or the presence of secondary phases. The XRD patterns correspond with the standard JCPDS file no. 01–080–1268, verifying the body–centered monoclinic structure within the C2/c space group. Rietveld refinement, an advanced method for detailed analysis of XRD data, was utilized. The derived lattice parameters, detailed in Table 1, substantiate the high-quality crystal structure, as reflected by the low R–factors and a goodness–of–fit (GOF) value of 2.32.

**Table 1:** Structural data obtained from the Rietveld refinement for CuO powder and sintered CuO ceramic.

Sample	CuO powder	Sintered CuO ceramic
a ($\dot{{\text{A}}}$)		
* a*	4.687	4.689
* b*	3.421	3.422
* c*	5.131	5.131
R_exp_ (%)	4.768	4.416
R_p_ (%)	4.284	4.476
R_wt_ (%)	6.135	6.727
GOF	1.655	2.321

**Figure 2:**
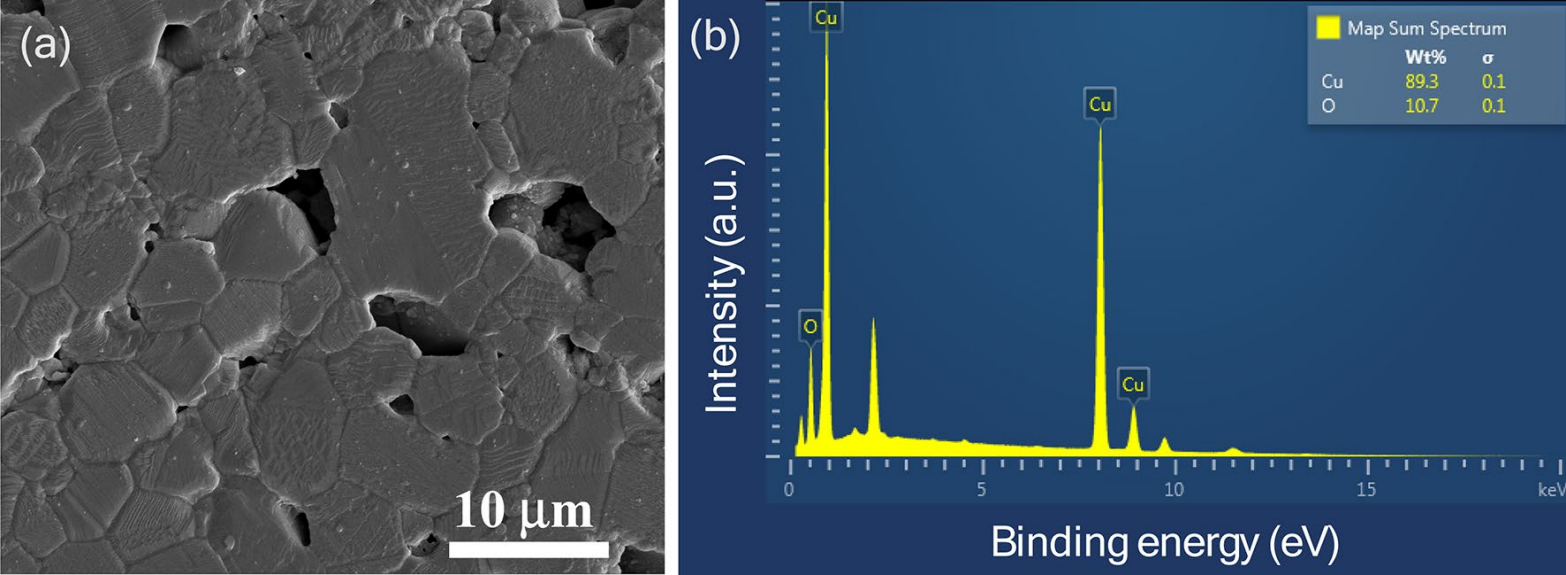
(**a**) SEM image and (**b**) EDS spectrum of sintered CuO ceramic.

Figure 2 illustrates the SEM image alongside an EDS spectrum of the sintered CuO ceramic. The SEM image displays a densely sintered microstructure with clearly defined grain boundaries, signifying a high degree of densification with a relative density of 88.9%. As reported by Li et al.^16^, the relative density of CuO sintered at 920 °C for 10 h was as low as ~ 55%. The presence of pores suggests that grain coarsening is prevalent, presenting a competitive dynamic to the sintering mechanism. The microstructure observed is consistent with those reported in prior studies^14,31,32^. The EDS spectrum offers an analysis of the elemental composition, verifying the presence of copper (Cu) and oxygen (O). Notably, the microstructural examination of sintered CuO ceramics through SEM is vital for understanding their electrical properties. The microstructure, characterized by densely packed grains and minimal porosity, is anticipated to enhance the performance of capacitive humidity sensors.

**Figure 3:**
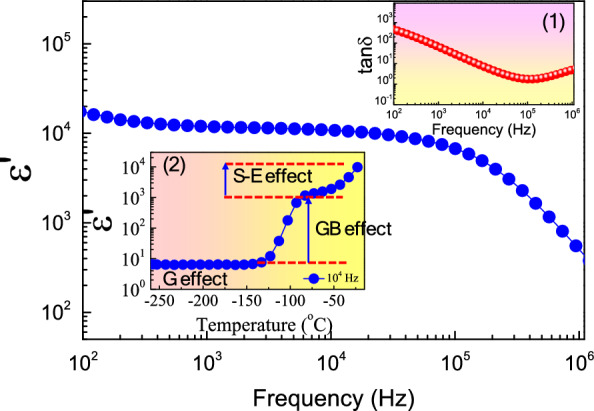
Dielectric permittivity (ε′) in frequency range of 10^2^–10^6^ Hz at ~ 25 °C for sintered CuO ceramic; insets (1) and (2) show loss tangent (tanδ) and temperature dependence of ε′ at 10^4^ Hz.

Figure 3 depicts the dielectric properties as a function of frequency at around 25 °C for sintered CuO ceramic. The material exhibits a remarkable dielectric response with a ε′ approximately 10^4^ over the frequency range of 10^2^ – 10^5^ Hz, consistent with values reported in earlier studies^12–16,31,32^. At higher frequencies, ε′ experiences a rapid decline, a phenomenon attributed to dielectric relaxation. In inset (1), the tanδ of CuO is observed to be significantly large (tanδ > 1.0 across the frequency spectrum) relative to other ceramics with high ε′ values, such as Mg–doped La_2-*x*_Sr_*x*_NiO_4_, CaCu_3_Ti_4_O_12_, TiO_2_, SrTiO_3_, and NiO–based compounds^4–6,10,17,19–22,28^. Therefore, the practical use of CuO materials in capacitors faces the significant hurdle of reducing the tanδ value to below 0.05. Further analysis into the comprehensive giant dielectric response of the sintered CuO ceramic was conducted at lower temperatures. As shown in inset (2) of Fig. 3, with a decrease in temperature to approximately − 80 °C, there is a reduction in ε′ from 10^4^ to 10^3^. This observation is likely due to the S–E effect, which is influenced by the choice of electrode materials^16^. At the interface between the semiconductive CuO surface and the metallic electrode, a non–Ohmic contact is established, leading to the formation of a Schottky barrier. This interface is subject to polarization when an electric field is applied. Consequently, the elevated ε′ recorded at temperatures above − 83 °C is primarily an artifact, resulting mainly from the S–E effect. At 10^4^ Hz, the ε′ at 25 °C is approximately 10,700, whereas at − 80 °C, it reduces to about 1150. It is thus deduced that the dielectric response associated with the S–E effect is in the realm of 10^4^.

Upon further cooling to approximately − 125 °C, the ε′ value sharply decreased from about 1150 to 12, suggesting that the dielectric response in this temperature range is on the order of 10^3^. This rapid decrease in ε′ is within the same magnitude as the decline observed for CaCu_3_Ti_4_O_12_ and related ACu_3_Ti_4_O_12_ oxides^3–5,17,25,26,28^, which is generally attributed to the IBLC effect. This effect could be due to the dielectric behavior of the grain boundaries or the IBLC effect itself. As the temperature dropped below − 125 °C, ε′ remained relatively stable, ranging between 6 and 10. At the lowest measured temperature of − 258 °C (15 K), the ε′ was 6.5, which is in line with literature values, specifically around 4.0 at 10 K for CuO sintered at 920 °C^16^. This indicates a dielectric response from ionic polarization, likely due to the displacement between Cu^2+^ and O^2−^ ions under an electric field. It is noteworthy that Li et al.^16^ reported an ε' value of approximately 110 at − 123 °C (150 K), which was ascribed to the IBLC effect at the grain boundary. However, in this study, at − 125 °C, the ε′ value was approximately 1150. The difference between these findings may be related to differences in microstructure. The CuO in the previous study had a porous structure with a relative density of about 55% (sintered at 920 °C), whereas in this study, a higher relative density of 88.9% (sintered at 980 °C) was achieved. Therefore, the IBLC effect, which pertains to the electrical behavior of the grain boundaries, should be more pronounced in this study due to the increased relative density, or in other words, a greater grain boundary density.

**Figure 4:**
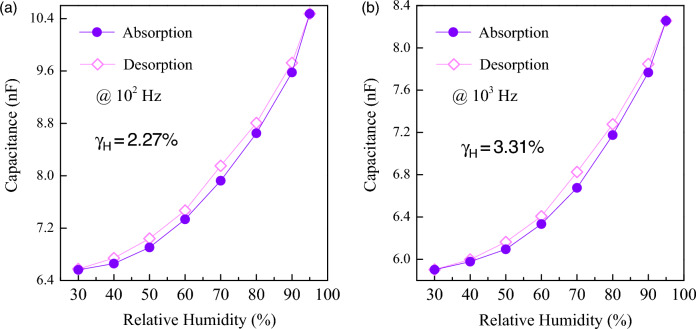
%RH dependence of capacitance (C_p_) at ~ 25 °C of sintered CuO ceramic at (**a**) 10^2^ and (**b**) 10^3^ Hz.

**Figure 5:**
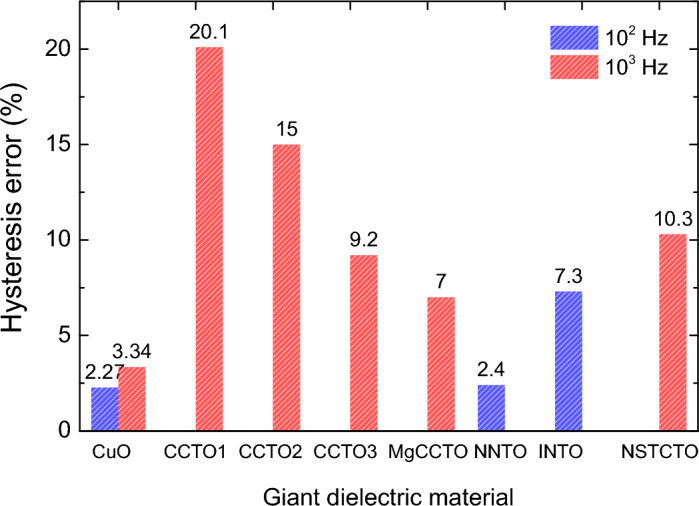
Comparison of hysteresis error at 10^2^ and 10^3^ Hz of CuO ceramic with many giant dielectric oxides: CCTO1, CCTO2, and CCTO3 are CaCu_3_Ti_4_O_12_ sintered 1100, 1050, and 1020 °C, respectively; MgCCTO is Mg–doped CCTO; NNTO is Na + Nb co–doped TiO_2_; INTO is In + Nb co–doped TiO_2_; and NSTCTO is (Na_1/3_Sr_1/3_Tb_1/3_)Cu_3_Ti_4_O_12_.

Figures 4a,b show the dependence of C_p_ on RH for sintered CuO ceramic at frequencies of 10^2^ Hz and 10^3^ Hz respectively. As clearly seen, it is apparent that the C_p_ increases with increasing RH for both frequencies. This indicates that the CuO ceramic is sensitive to changes in humidity, with its capacitive properties enhancing as the RH level rises. This is a typical behavior for materials that can be used for humidity sensing, as the presence of water molecules tends to increase the ε′ and, consequently, the C_p_ of the material. Notably, the results also show a slim hysteresis in the C_p_ measurements during absorption (humidifying process) and desorption (drying process). Hysteresis in humidity sensors is the difference in capacitance response for the same RH level during increasing and decreasing moisture conditions^26,27^. Lower hysteresis is generally desirable for more accurate and reliable humidity measurements. The γ_H,max_ can be calculated from the Equation^26^.:

1 $${\upgamma }_{{{\text{H}},{\text{max}}}} = \frac{{{\Delta H}_{{{\text{max}}}} }}{{2{\text{F}}_{{{\text{FS}}}} }},$$

where F_FS_ is the full–scale C_p_ differential between 30% RH and 95% RH, and ΔH_max_ is the maximum C_p_ difference observed between the absorption and desorption curves. The maximum hysteresis error (γ_H,max_) of 2.27% at 10^2^ Hz and 3.31% at 10^3^ Hz were obtained. The results corroborate the overview provided, indicating that CuO ceramic has promising characteristics for use in humidity sensors, with a relatively low hysteresis error. The γ_H,max_ values at 10^2^ and 10^3^ Hz of the CuO ceramic are comparable to those previous reports for bulk ceramics, as illustrated in Fig. 5. Notably, the γ_H,max_ values are lower than those of other materials like CaCu_3_Ti_4_O_12_^26^, (Na_1/3_Sr_1/3_Tb_1/3_)CaCu_3_Ti_4_O_12_^28^, and co–doped TiO_2_ ceramics^23,27^. The calculated relative density of CuO is 88.9%, resulting in a porosity of 11.1%. Porosity is one of the most important factors that contribute to the overall humidity sensing properties of dielectric oxides. Therefore, the significantly enhanced humidity sensing properties of CuO can be attributed to its porosity. It is worth noting that CuO stands out for its chemical stability, cost-effectiveness, and environmental friendliness, offering long-lasting, accurate humidity sensing without frequent recalibration. Its synthesis is straightforward and economical, promoting its integration into compact devices for widespread commercial use.

Additionally, it is important to highlight that the measured C_p_ exhibited a slight increase with a rise in humidity from 30% RH to 50% RH. The ε′ recorded in Fig. 3, measured at a temperature of approximately 25 °C in a closed room with air conditioning set to around 50% RH, was 11,887 at 10^4^ Hz. Conversely, the ε′ in the inset (2) of Fig. 3, measured in a closed–cycle helium cryostat, was 9,801 at the maximum temperature point for the same frequency. This slight discrepancy in ε′ values between the two settings aligns with the observations in Fig. 4, where ε′ modestly increases as the humidity rises from 30% RH to 50% RH.

**Figure 6:**
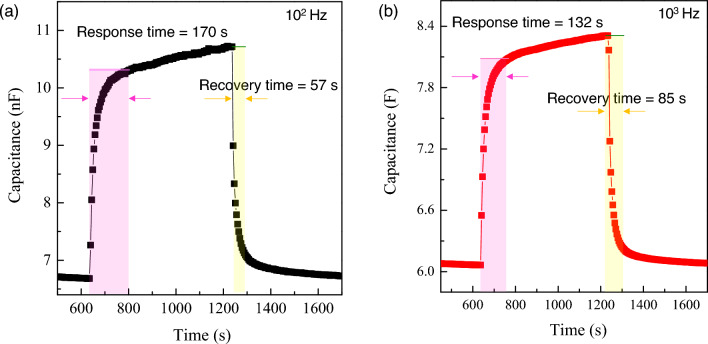
Response and recovery times of sintered CuO ceramic at (**a**) 10^2^ and (**b**) 10^3^ Hz.

To further explore the potential use of CuO in capacitive humidity sensors, it is crucial to consider the response and recovery times, which are key parameters of humidity sensing materials^29^. The response time is the period required for the sensor to reach 90% of the total change during moisture adsorption, while the recovery time is the duration needed to achieve 90% of the total change during moisture desorption. In Fig. 6, we examine the dynamic humidity sensing behavior of sintered CuO ceramics at two different frequencies: 10^2^ Hz and 10^3^ Hz. At 10^2^ Hz, the CuO ceramic sensor demonstrates a response time of approximately 2.8 min (170 s), quickly adjusting to elevated humidity levels, and a recovery time of around 1 min (57 s), denoting a swift return to base–level capacitance following a humidity reduction. In contrast, at 10^3^ Hz, the sensor responds more promptly with a response time of approximately 2.2 min (132 s) and a slightly longer recovery time of around 1.4 min (85 s). Notably, the response and recovery times for CaCu_3_Ti_4_O_12_ and Mg–doped CaCu_3_Ti_4_O_12_ range from 13 to 30 min^26^. For (Na, Nb) co–doped TiO_2_, the response and recovery times are reported at 1.9 and 0.3 min, respectively^23^. Similarly, for (Na, Nb) co–doped TiO_2_^22^, the figures are 2.75 and 0.5 min. (Na_1/3_Sr_1/3_Tb_1/3_)Cu_3_Ti_4_O_12_ exhibited response and recovery times of 1.8 and 3.7 min, respectively^28^. Therefore, CuO emerges as a particularly promising bulk humidity-sensing material, distinguished by its low γ_H,max_ and notably brief response and recovery times.

**Figure 7:**
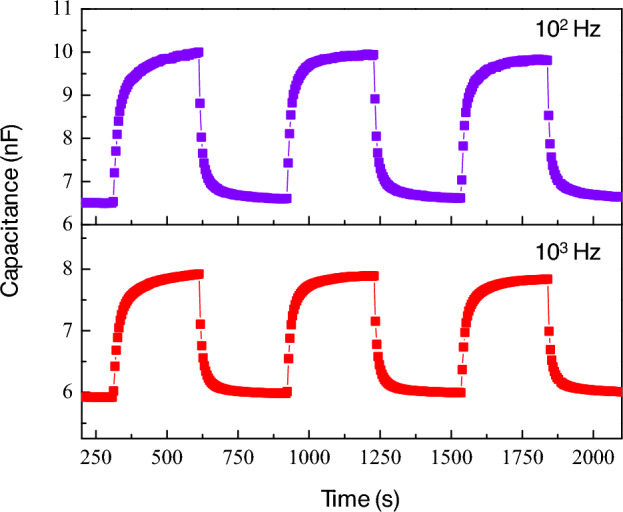
Repeatability of humidity sensing response and recovery for sintered CuO ceramic at 10^2^ and 10^3^ Hz.

Figure 7 demonstrates the repeatability of humidity sensing by sintered CuO ceramics at frequencies of 10^2^ Hz and 10^3^ Hz. The C_p_ values exhibit consistent cyclical changes between distinct high and low states, indicative of the sensor’s stable performance over multiple humidity exposure cycles. This consistent repeatability is essential for the reliability of the sensor in applications that demand accurate and repeated humidity measurements. Delving deeper into the discussion, the ability of the sensor to consistently return to its baseline capacitance after each cycle suggests a limited degree of hysteresis, a valuable characteristic in sensing materials. The consistent response of the CuO ceramic sensor at both tested frequencies suggests that it maintains sensitivity across a range of operational conditions. This characteristic highlights the versatility of the material in adapting to diverse environmental scenarios where humidity levels may fluctuate rapidly and unpredictably. Moreover, the clear distinction between the high and low humidity states across multiple cycles accentuates the accuracy of the sensor in detecting changes in humidity, reinforcing the suitability of CuO ceramics for integration into sophisticated sensing technologies.

In research on capacitive humidity–sensing oxides, it has been proposed that when exposed to humid air, these oxides initially chemically adsorb a small amount of H_2_O molecules on both their outer surface and the inner surfaces of grains in porous ceramic oxides^33^. This leads to the dissociation of the H_2_O molecules into two hydroxyl ions. Following this, a complete layer of surface hydroxyl groups is established, enabling further physical adsorption of H_2_O molecules. At this initial phase, the physisorbed layer is distinguished by the double hydrogen bonding of individual H_2_O molecules. As RH continues to rise, the adsorption shifts from a single–layer to a multi–layer arrangement, with the H_2_O molecules in subsequent layers being singly bonded. Notably, the hydroxyls in the chemisorbed layer start to desorb at around 400 °C^34^. Thus, the notable changes in the C_p_ of CuO ceramics due to RH variations are mainly due to the H_2_O molecules physically adsorbed on the outer surfaces and/or within the grain boundaries, which have considerable dielectric properties^26^. In the case of CaCu_3_Ti_4_O_12_–based materials^26,28,33^, the adsorption of H_2_O molecules at grain boundaries enhances the C_p_ of these boundaries by promoting increased charge accumulation. This effect elevates the ε′ or C_p_, especially at lower frequencies.

When the CuO ceramic was subjected to alternating current electric fields during dielectric measurements, the associated dipole moments of the physically adsorbed H_2_O molecules aligned with the direction of the electric field, resulting in increased polarization intensity and an elevated capacitance C_p_ value^35^. As shown in Fig. 4, an increase in humidity from 30 to 50% RH resulted in a slight increase in C_p_, attributable to a small number of physisorbed H_2_O molecules. Most H_2_O molecules are chemically adsorbed, which have only a slight effect on the dielectric response. However, as the humidity increased from 50 to 95% RH, the number of physisorbed H_2_O molecules continuously rose with the increasing humidity. This led to an enhanced polarization intensity, thereby boosting the dielectric response and C_p_ values. It is worth noting that at the higher frequency of 10^3^ Hz, the direction of the electric field changes more rapidly compared to the lower frequency of 10^2^ Hz. This facilitates a greater accumulation of polarization, largely due to the orientation of water molecules. In exploring the origins of the giant dielectric response in CuO ceramics, the IBLC and S–E effects have been identified as key contributors to the observed high ε′ at temperatures above − 100 °C. Hence, the humidity sensing properties may primarily stem from two mechanisms. The first mechanism concerns the grain boundary effect, where the adsorption of water molecules at grain boundaries notably enhances the C_p_ of these areas. This enhancement comes through the increased accumulation of OH^–^ and H_3_O^+^ ions, similar to the behavior seen in CaCu_3_Ti_4_O_12_–based compounds. The second mechanism relates to the S–E effect. Per Schottky–Mott theory^16^, the Schottky barrier height (Φ_b_) for ideal electrical contacts is exclusively determined by the disparity between the work function of the metal electrode and the electron affinity of the semiconductor. Krohns et al.^36^ have illustrated that the low–frequency giant dielectric properties of ceramic oxides are significantly influenced by surface treatments prior to the creation of parallel electrodes, a process crucial for the formation of a Schottky barrier at the contact–bulk interface. Experiments with Ag paint electrodes have shown that large air gaps at the interface for silver–paint contacts result in a less effective establishment of Schottky barriers^37,38^. Moreover, interface states, including interfacial bonding, surface defects, oxygen adsorption, and contamination, play a critical role in affecting Φ_b_^39^. Consequently, the adsorption of water molecules can alter Φ_b_, inducing a dielectric response and, thus, impacting C_p_.

## Conclusions

In this study, we successfully demonstrated the effective use of CuO ceramics as a potential material for humidity sensing applications. Our approach involved the fabrication of CuO samples through a sintering process at 980 °C for 5 h, achieving a microstructure with a relative density of 88.9% and pure phase. One of the most significant findings was the observation of giant dielectric properties in CuO at room temperature, attributable to extrinsic factors such as IBLC effects and S–E contact phenomena. Our experiments focused on the behavior of these dielectric properties under varying levels of RH, specifically at frequencies of 10^2^ and 10^3^ Hz. A continuous increase in capacitance was noted as RH levels rose from 30 to 95%, demonstrating the sensitivity to humidity changes related to the IBLC and S–E effects. Moreover, the study revealed a minimal hysteresis error, with a maximum of 2.3% at 10^2^ Hz and 3.3% at 10^3^ Hz, underscoring the precision of CuO ceramics in humidity detection. The response and recovery times were remarkably quick, ~ 2.8 min and 0.95 min, respectively, which is advantageous for real–time sensing applications. Additionally, the humidity response was repeatable, indicating the reliability of CuO ceramics in consistent performance. Overall, our research highlights the substantial potential of CuO as a giant dielectric material suitable for humidity sensor applications.

## Data Availability

The data of this study are available from the corresponding author upon reasonable request.

## References

[1] Wang Y, Jie W, Yang C, Wei X, Hao J (2019). Colossal permittivity materials as superior dielectrics for diverse applications. Adv. Funct. Mater..

[2] Yang L (2023). Synthetic technologies, property enhancements and versatile applications of calcium copper titanate: A review. Nano Energy.

[3] Gecil Evangeline T, Raja Annamalai A, Ctibor P (2023). Dielectric response and low dielectric loss of gadolinium-doped CaCu_3_Ti_4_O_12_ ceramics processed through conventional and microwave sintering. J. Electron. Mater..

[4] Hao W, Xu P, Han P, Wang M (2023). Optimize the dielectric properties of CaCu_3_Ti_4_O_12_ ceramics by adjusting the conductivities of grains and grain boundaries. J. Eur. Ceram. Soc..

[5] Jesus LM, Barbosa LB, Ardila DR, Silva RS, M'Peko JC (2023). Electrical microstructure evolution of CaCu_3_Ti_4_O_12_ (CCTO) ceramics: From resistive and core shell-like to semiconducting grains. Ceram. Int..

[6] Xie H (2023). Suppressing resistance degradation in SrTiO_3_-based colossal permittivity capacitor material. Ceram. Int..

[7] Chen Y, Zeng Y, Cao W, Chen N, Du G (2022). Colossal permittivity and low dielectric loss in (Li, Nb) co-doped SrTiO_3_ ceramics with high frequency and temperature stability. Ceram. Int..

[8] Zhang X (2022). Simultaneously achieving colossal permittivity, ultralow dielectric loss tangent, and high insulation resistivity in Er-doped SrTiO_3_ ceramics via oxygen vacancy regulation. ACS Appl. Mater. Interfaces.

[9] Meeporn K, Chanlek N, Thongbai P (2016). Effects of DC bias on non-ohmic sample-electrode contact and grain boundary responses in giant-permittivity La_1.7_Sr_0.3_Ni_1-x_Mg_x_O_4_ ceramics. RSC Adv..

[10] Xue Y (2023). Colossal permittivity and low loss in (In_0.5_Ta_0.5_)_0.1_Ti_0.9_O_2_ ceramics with a stable temperature range of X9D. J. Mater. Sci. Mater. Electron..

[11] Guo X (2023). Defect chemistry and optimized dielectric properties of Ni_1-δ_O ceramics via doping donor ions. Ceram. Int..

[12] Sarkar S, Jana PK, Chaudhuri BK, Sakata H (2006). Copper (II) oxide as a giant dielectric material. Appl. Phys. Lett..

[13] Sarkar S, Jana PK, Chaudhuri BK (2008). Colossal internal barrier layer capacitance effect in polycrystalline copper (II) oxide. Appl. Phys. Lett..

[14] Thongbai P, Maensiri S, Yamwong T (2008). Effects of grain, grain boundary, and dc electric field on giant dielectric response in high purity CuO ceramics. J. Appl. Phys..

[15] Thongbai P, Yamwong T, Maensiri S (2008). Correlation between giant dielectric response and electrical conductivity of CuO ceramic. Solid State Commun..

[16] Li M, Feteira A, Sinclair DC (2009). Relaxor ferroelectric-like high effective permittivity in leaky dielectrics/oxide semiconductors induced by electrode effects: A case study of CuO ceramics. J. Appl. Phys..

[17] Jalafi I (2023). High permittivity and low dielectric loss of the (Ca_0.9_Sr_0.1_)_1-x_La_2x/3_Cu_3_Ti_4_O_12_ ceramics. Ceram. Int..

[18] Liu J, Liu Q, Zhu P (2023). Dielectric relaxations without colossal permittivity in Mg and Nb co-doped BaTiO_3_ ceramics. Ceram. Int..

[19] Meeporn K, Chanlek N, Srepusharawoot P, Thongbai P (2023). Extremely reduced loss tangent with retaining ultra high dielectric permittivity in Mg^2+^-doped La_1.9_Sr_0.1_NiO_4_ ceramics. Heliyon.

[20] Wang M, Xie J, Xue K, Li L (2022). Tuning dielectric properties in a rutile TiO_2_ system via synergistic design of surface structure and microstructure. Appl. Surf. Sci..

[21] Ma J-J, Gao Y, Chen Y, Wang M-H (2023). Effects of (Ag, Ta) doping on the microstructure and dielectric properties of titanium dioxide functional ceramics. J. Electron. Mater..

[22] Si RJ (2019). Humidity sensing behavior and its influence on the dielectric properties of (In + Nb) co-doped TiO_2_ ceramics. J. Mater. Sci..

[23] Li T (2019). Microstructure, colossal permittivity, and humidity sensitivity of (Na, Nb) co-doped rutile TiO_2_ ceramics. J. Am. Ceram. Soc..

[24] Wang J (2019). The effect of humidity on the dielectric properties of (In + Nb) co-doped SnO_2_ ceramics. J. Eur. Ceram. Soc..

[25] Li M (2016). Study of the humidity-sensing mechanism of CaCu_3_Ti_4_O_12_. Sens. Actuators B Chem..

[26] Li M, Chen XL, Zhang DF, Wang WY, Wang WJ (2010). Humidity sensitive properties of pure and Mg-doped CaCu_3_Ti_4_O_12_. Sens. Actuators B Chem..

[27] Li TY (2019). Giant and controllable humidity sensitivity achieved in (Na+Nb) co-doped rutile TiO_2_. Sens. Actuators B Chem..

[28] Srilarueang S, Putasaeng B, Sreejivungsa K, Thanamoon N, Thongbai P (2023). Giant dielectric response, nonlinear characteristics, and humidity sensing properties of a novel perovskite: Na_1/3_Sr_1/3_Tb_1/3_Cu_3_Ti_4_O_12_. RSC Adv..

[29] Ganbold E (2023). Highly sensitive interdigitated capacitive humidity sensors based on sponge-like nanoporous PVDF/LiCl composite for real-time monitoring. ACS Appl. Mater. Interfaces.

[30] Tachibana S (2022). A printed flexible humidity sensor with high sensitivity and fast response using a cellulose nanofiber/carbon black composite. ACS Appl. Mater. Interfaces.

[31] Putjuso T, Manyum P, Yamwong T, Thongbai P, Maensiri S (2011). Effect of annealing on electrical responses of electrode and surface layer in giant-permittivity CuO ceramic. Solid State Sci..

[32] Putjuso T (2011). Giant dielectric behavior of solution-growth CuO ceramics subjected to dc bias voltage and uniaxial compressive stress. Solid State Sci..

[33] Chattopadhyay A, Nayak J (2022). Improvement of humidity sensing performance and dielectric response through pH variation in CaCu_3_Ti_4_O_12_ ceramics. Sens. Actuators A Phys..

[34] Egashira M, Nakashima M, Kawasumi S, Selyama T (1981). Temperature programmed desorption study of water adsorbed on metal oxides. 2. Tin oxide surfaces. J. Phys. Chem..

[35] Kao K-C (2004). Dielectric Phenomena in Solids: With Emphasis on Physical Concepts of Electronic Processes.

[36] Krohns S, Lunkenheimer P, Ebbinghaus SG, Loidl A (2008). Colossal dielectric constants in single-crystalline and ceramic CaCu_3_Ti_4_O_12_ investigated by broadband dielectric spectroscopy. J. Appl. Phys..

[37] Lunkenheimer P (2002). Origin of apparent colossal dielectric constants. Phys. Rev. B.

[38] Lunkenheimer P, Fichtl R, Ebbinghaus S, Loidl A (2004). Nonintrinsic origin of the colossal dielectric constants in CaCu_3_Ti_4_O_12_. Phys. Rev. B.

[39] Brillson LJ (2007). Interface bonding, chemical reactions, and defect formation at metal-semiconductor interfaces. J. Vac. Sci. Technol. A.

